# Applying Field and Genomic Epidemiology Methods to Investigate Transmission Networks of Highly Pathogenic Avian Influenza A (H5N1) in Domestic Poultry in British Columbia, Canada (2022–2023)

**DOI:** 10.1155/tbed/4099285

**Published:** 2025-07-15

**Authors:** Krista Howden, Shannon K. French, Manon Racicot, Anthony V. Signore, Caitlyn Best, Jacklyn Perrey, Troy Bourque, Yohannes Berhane

**Affiliations:** ^1^Canadian Food Inspection Agency, Calgary, Alberta T2E 9B2, Canada; ^2^Canadian Food Inspection Agency, Guelph, Ontario N1G 4S9, Canada; ^3^Canadian Food Inspection Agency, Saint-Hyacinthe, Quebec J2S 2M2, Canada; ^4^National Centre for Foreign Animal Disease, Winnipeg, Manitoba R3E 3M4, Canada; ^5^Canadian Food Inspection Agency, Parksville, British Columbia V9P 1V7, Canada; ^6^Canadian Food Inspection Agency, Abbotsford, British Columbia V2T 6W3, Canada; ^7^Department of Veterinary Pathology, Western College of Veterinary Medicine, University of Saskatchewan, Saskatoon S7N 5B4, Canada; ^8^Department of Animal Science, University of Manitoba, Winnipeg, Manitoba R3T 2N2, Canada; ^9^Department of Pathobiology, University of Guelph, Guelph, Ontario N1G 2W1, Canada

## Abstract

Highly pathogenic avian influenza (HPAI) virus A (H5N1) was first detected in North America in 2021. Ongoing spillover events from wild to domestic birds and subsequent transmission between domestic birds resulted in Canada's largest recorded epidemic of HPAI. Between December 2021 and April 2024, 422 A (H5N1) outbreaks in domestic birds were confirmed in Canada. Of these, 158 (37%) occurred in British Columbia (BC). This study integrates field and genomic epidemiology (GE) results to investigate the potential for lateral or local spread between infected poultry farms in BC. Five genetically distinct viral clusters were identified during this period. Among the 31 noncommercial premises, 27 (87.1%) were classified as independent introductions, while four premises (12.9%) were attributed to local spread within 10 km of a phylogenetically connected infected premises (IP). No lateral spread events were identified among noncommercial premises. All infected noncommercial premises housed birds with outdoor access, emphasizing their susceptibility to wild bird exposure. Of the 127 infected commercial poultry premises, 21 (16.5%) were classified as independent introductions, 82 (64.6%) as local spread, 18 (14.2%) with potential for lateral spread, five (3.9%) with potential for both local and/or lateral spread, and one (0.8%) for which sequencing was unavailable. Local spread emerged as a prominent feature, with most IP in proximity to one another having genetically similar viruses. Results suggest that proximity (<200 m) to an IP was a more reliable predictor of future infection status than contact with an IP. These findings underscore the critical value of combining field and GE to understand outbreak dynamics comprehensively. This integrative approach improves resource allocation, informs targeted containment strategies, and supports the need for effective biosecurity measures to mitigate future risks, particularly in densely populated poultry production regions. Robust interventions are needed to address both independent introductions and secondary spread pathways.

## 1. Introduction

The first outbreak of highly pathogenic avian influenza (HPAI) virus due to A/goose/Guangdong/1/1996 lineage (Gs/Gd) 2.3.4.4b clade A (H5N1) virus was detected in St. John's, Newfoundland (NL), Canada, in December 2021 following a mortality event in domestic birds. Retrospective analyses revealed the presence of the virus in a great black-backed gull from the same area earlier. Genetic analysis suggested that this virus had emerged from Northwest Europe, likely dispersing from Europe in late winter or early spring of 2021 and reaching NL in the fall of 2021, carried across the Atlantic by migratory birds. The virus isolated from the gull differed from that found in domestic birds by only a few nucleotide differences in the neuraminidase (*N*) gene segment. This marked the first presence of Gs/Gd lineage HPAI H5 in the Americas since June 2015 [1].

In addition to the A (H5N1) virus detected in the NL gull, a phylogenetically distinct clade 2.3.4.4b A (H5N1) virus was detected in a moribund bald eagle in British Columbia (BC) in February 2022. This virus was genetically related to a strain isolated in Hokkaido, Japan, in January 2022, indicating that it was introduced to Canada via the Pacific flyway. These findings suggest multiple transcontinental introductions of A (H5N1) through migratory wild birds [2].

Following these detection events, Canada's Interagency Surveillance Program for Avian Influenza in Wild Birds identified A (H5N1) in all Canadian provinces and territories. Wild Anseriformes (ducks and geese) and Charadriiformes (gulls, terns, and other shorebirds) serve as the primary reservoirs for influenza A viruses, including A (H5N1), and their movements are responsible for translocating the virus across Canada [3]. In addition to the original Eurasian strains identified on Canada's Atlantic and Pacific coasts, whole genome sequencing (WGS) identified multiple reassortments of mixed Eurasian and North American (NA) origins distributed across the country [4].

Significant morbidity and mortality were observed in wild birds, which served as a reservoir for domestic birds. Ongoing spillover events from wild to domestic birds and subsequent transmission between domestic birds resulted in Canada's largest recorded event of HPAI. Between December 2021 and April 2024, 422 A (H5N1) outbreaks in domestic birds were confirmed in Canada. Of these, 158 occurred in BC between April 2022 and December 2023. Initial phylogenetic data suggested multiple spillover events from wild birds to poultry and could not rule out transmission between farms [5]. The outbreaks in BC included 127 commercial poultry premises, 19 noncommercial smallholder poultry premises, and 12 noncommercial nonpoultry premises. The outbreaks in BC accounted for 37.4% of Canada's domestic bird outbreaks and 42.9% of the commercial poultry outbreaks. BC's commercial poultry industry accounted for approximately 12% of Canada's total production in 2023 [6], so the number of A (H5N1) cases in BC's commercial poultry premises was disproportionately high.

The poultry sector is economically significant in BC, with premises production sales of $657 million in 2023 [6]. BC has approximately 580 regulated commercial poultry producers, with four supply-managed commodities: chicken (meat), layers (eggs), broilers, and turkeys [7]. Smaller commercial sectors include layer pullet growers, layer breeders, and other poultry, such as ducks, geese, squabs (pigeons), pheasants, quail, and silkie chickens. Small flocks are common on acreages throughout BC, often including rare or specialty poultry breeds or other species like pheasants, ducks, geese, or other game birds. The poultry housing, handling, and care standards in BC are set by respective marketing boards, with minimum standards enforced by the National Farm Animal Care Council (NFACC). The National Avian On-Farm Biosecurity Standard, developed in collaboration with provincial/territorial governments, academia, and industry, forms the basis of a comprehensive voluntary program designed to provide applicable guidance for owners or managers of all poultry production types in Canada. It has been developed as a tool for all people and businesses handling and keeping poultry, including large-scale supply-managed producers, backyard flock owners, and other domestic bird keepers [8]. A BC Poultry Biosecurity Reference Guide informed by the National standards was developed in 2006 as part of the BC Poultry Biosecurity Program [9]. The program allowed producers to plan and implement biosecurity practices that meet mandatory biosecurity standards for commercial birds as required by the relevant Boards and Commission. All supply-managed producers are subject to regular audits of their on-premises animal welfare standards, biosecurity, and food safety programs [10]. Although not mandatory for nonsupply-managed poultry, the province also provides guidance to encourage biosecurity practices [11].

BC has experienced past HPAI outbreaks, underscoring the region's vulnerability and the challenges of maintaining consistent high-level biosecurity. These historical outbreaks demonstrate the pathways through which wild birds can introduce the virus to domestic flocks. Implementing effective measures to prevent wild bird access and indirect contact with a contaminated environment in poultry facilities can be challenging due to the natural and agricultural landscape, increasing the risk of HPAI introduction. The first NA outbreak of Eurasian Gs/Gd H5N2 and H5N1 virus occurred in BC's Fraser Valley (FV) in late November 2014. During this event, 11 commercial and one noncommercial smallholder premises were infected. The epidemiological investigation identified indirect contact with wild birds as the most likely source for four (36%) commercial infected premises (IP) and the single noncommercial premises (100%). Lateral spread between premises via the movement of birds or service providers was suspected as the source for three (25%) premises, while localized/environmental spread was suspected for the remaining four (33%) premises. Genomic epidemiology (GE) results largely supported the transmission network inferred by the field epidemiology (FE) analysis [12]. This previous outbreak demonstrated that migratory birds could introduce HPAI. With a continual risk of introduction from infected wild birds and/or a contaminated environment, differentiating between a wild bird or other domestic bird source through premises-to-premises transmission is challenging.

Field and GE are complementary approaches to investigating disease outbreaks, offering unique insights and advantages. Combining the results of both methods can significantly enhance our understanding of the source of disease introduction and potential disease spread events. For avian influenza (AI) outbreaks in domestic poultry, employing both approaches is critical, given the complex interrelationship between wild and domestic birds, humans, and mammals, which significantly influences the dynamics of disease spread and the inherent limitations of each approach.

Influenza A viruses are prime candidates for combining field and GE due to their propensity for genetic shift and drift during RNA replication. These mechanisms of viral evolution can both complicate and inform outbreak investigations. Antigenic drift involves minor genetic mutations that accumulate over time in the virus's RNA due to the error-prone nature of RNA polymerase. These gradual changes can alter the virus's surface proteins (hemagglutinin and neuraminidase), leading to new virus variants. A more dramatic change occurs when the virus acquires new segments from different influenza A strains, often through reassortment, resulting in a novel virus with significantly different antigenic properties [13].

CFIA FE involves directly investigating disease occurrences in populations through questionnaires, interviews, and premises visits. This approach focuses on describing an outbreak in terms of animal, time, and space. By identifying and analyzing patterns, hypotheses on risk and protective factors, potential sources of disease introduction, and transmission opportunities can be generated. FE provides detailed contextual information about the outbreak, such as common ownership or management, premises, and service provider biosecurity practices, animal and fomite movement events, and environmental factors. FE can enable rapid identification of potential sources and transmission routes through direct observation, interviews, and associated case-finding via follow-up tracing investigations. However, FE has limitations, the most significant being the reliance on producer-reported data, which can be biased, inaccurate, or incomplete. Additionally, it may have limited resolution, as not all transmission pathways or subtle links between cases may be identified.

GE leverages genome sequencing to study the spread and evolution of pathogens within populations. By co-analyzing epidemiological data with the genetic sequences of pathogens (phylodynamics), it is possible to infer transmission patterns, identify genetically related viruses between premises, track the emergence of new variants, and monitor how immunity, intervention strategies, and population structure influence its spread [14].

GE provides a powerful toolkit for real-time monitoring and responding to infectious disease outbreaks. This approach has high resolution and offers precise information about specific pathogen strains and their relationships, enabling the identification of specific evolutionary pathways and potential connections between cases. However, GE requires advanced technology, specialized skills, and significant financial investment. Sample processing and analysis can be time-consuming, and delayed results can preclude real-time decision-making during an outbreak. Moreover, in cases where a virus spreads rapidly across premises, there is little opportunity for single-nucleotide polymorphisms (SNPs) to accumulate. In these instances, GE is often unable to estimate the direction of viral spread with high confidence.

The unprecedented scale and resource demands of the A (H5N1) epidemic in BC in 2022 and 2023 significantly impacted individual producers, the broader poultry industry, and the Canadian Food Inspection Agency's (CFIA) response. CFIA was on-site for biocontainment, destruction, and cleaning/disinfection activities; however, due to the magnitude and duration of this event and the ongoing COVID-19 pandemic, on-site FE investigations were not feasible for most premises, and resources were focused on tracing and analysis. This study used the available field and GE results from the 158 IP in BC between 2022 and 2023 to better understand the viral origin and transmission pathways. A secondary aim was to identify processes by which available FE and GE tools can be utilized stepwise to optimize epidemiological resources for future outbreaks.

## 2. Materials and Methods

### 2.1. Field Epidemiological Investigation Methods and Tools

Case finding using active dead bird surveillance within primary control zones (PCZs), follow-up investigation, and testing of birds on epidemiologically linked premises and premises reporting sick birds were completed. Oropharyngeal and cloacal swabs were taken from dead or moribund domestic birds and tested at BC's Animal Health Centre, a Canadian Animal Health Surveillance Network (CAHSN) laboratory. CAHSN is a network of federal, provincial, and university animal health laboratories across Canada collaborating to provide improved early detection and rapid response to animal disease threats and minimize risks to animal health, public health, and Canada's economy. Samples that tested positive for H5/H7 were then sent for confirmatory testing and WGS by the National Center for Foreign Animal Disease (NCFAD) in Winnipeg, Canada, as described previously [4]. Each location on which A (H5N1) was detected and confirmed by the NCFAD was declared as an IP. IP were numbered sequentially based on the initial detection date at BC Animal Health Centre or NCFAD.

National clusters of IP in time were classified by outbreak “wave” coinciding with the spring and fall wild bird migration period of each year. The start dates of each wave were determined based on the date clinical signs were first observed on the initial IP of each period across Canada: December 9th, 2021, for “Wave 1”; August 30, 2022, for “Wave 2”; February 25th, 2023, for “Wave 3”; and September 9th; 2023 for “Wave 4”.

Each IP was issued a declaration of infected place and a requirement to quarantine by the CFIA, the competent veterinary authority in Canada, to restrict the movement of people, birds, and potential fomites [15]. Each IP was first classified according to the WOAH [16] definitions of poultry and nonpoultry. CFIA further categorized premises according to the level of risk of virus spread by applying a risk categorization aid [17]. This risk-based approach leads to three classifications for each IP: commercial poultry, noncommercial poultry (smallholder), or noncommercial nonpoultry.

Up to November 2023, for the purpose of CFIA's disease response, commercial refers to supply-managed premises or premises with 300 or more birds, while noncommercial describes premises with less than 300 birds. As of November 9^th^, 2023, commercial poultry refers to birds raised under Canada's supply management (quota) system for producing or selling their products or breeding for these purposes or birds raised outside the quota system, on a premises with either (1) 1000 or more birds on-site in total, for producing or selling their products or breeding for these purposes, or (2) 300 or more Anseriformes for producing or selling their products or breeding for these purposes. Noncommercial poultry is defined as birds raised in smaller flocks of fewer than 1000 birds total and fewer than 300 Anseriformes, for producing or selling their products locally for limited sales or for breeding for these purposes [17].

Poultry means all birds reared or kept in captivity for the production of any commercial animal products or for breeding for this purpose, fighting cocks used for any purpose, and all birds used for restocking supplies of game or for breeding for this purpose, until they are released from captivity. Birds that are kept in a single household, the products of which are used within the same household exclusively, are not considered poultry, provided that they have no direct or indirect contact with poultry or poultry facilities. Birds that are kept in captivity for other reasons, including those that are kept for shows, racing, exhibitions, zoological collections and competitions, and for breeding or selling for these purposes, as well as pet birds, are not considered poultry, provided that they have no direct or indirect contact with poultry or poultry facilities [18].

The initial premises-level epidemiological information was collected in CFIA's Avian Influenza Premises Investigation Questionnaire (PIQ) [17]. This questionnaire is specific to avian species and incorporates event-specific directions for the A (H5N1) response starting in 2022. This tool captures information on the premises, such as the start date (date of first clinical signs) and location of birds with clinical signs or increased mortality, the species and type of production, the number of clinical and susceptible birds, the site plan, and biosecurity practices in place (management of feed, people, vehicles, equipment, wildlife, deadstock, etc.). As per WOAH's [16] guidance on responding to HPAI, movement event data is collected for all people, poultry, poultry products, and things exposed to poultry or poultry products associated with an IP during the 21 days before the onset of clinical signs of AI.

The tracing period is when all movements on or off IP must be investigated as possible sources (trace-in period) or spread (trace-out period) of the virus. It starts on the earliest possible date that the disease agent could have been introduced and continues up to and including the day when movement controls were put in place [19]. The date of first clinical signs on each IP was determined by producer interview and examination of production records (i.e., mortality, feed, and water consumption), where available. The purpose of epidemiological tracing is to identify potential sources of introduction of AI virus to IP and to identify premises at risk of being exposed to AI virus by direct or indirect contact with an IP [17].

All direct poultry movements on and off an IP within the tracing period were immediately investigated as high-risk contacts. Trace-in (onto premises) and trace-out (off premises) premises with no confirmed direct contact between birds were subject to a qualitative risk assessment to determine indirect transmission potential. Indirect movements refer to exposure to the HPAI virus via contaminated environments, equipment, or personnel without physical interaction between infected and susceptible birds. The level of risk varies based on the contamination level and the route of exposure and is classified as moderate, low, or negligible risk. Moderate risk contacts include the movement of people and associated fomites in contact with live birds at the source and destination (e.g., shared personnel, catching crews, and feed specialists). Low-risk indirect contacts include situations where the potential for transmission exists but is limited due to biosecurity measures or reduced contamination levels (e.g., brief vehicle exposure to contaminated areas with some biosecurity measures in place). Negligible risk indirect contacts include situations where the likelihood of transmission is extremely low due to robust biosecurity measures, minimal virus survival, or lack of effective exposure routes. All high-risk contact premises (HRCP) were issued a Declaration of Infected Place and a Requirement to Quarantine that remained in place until 14 days after the last date of contact with the IP and all associated surveillance activities had been completed with negative results [17].

Given the substantial number of IP detected weekly in the fall of 2022 and 2023 and the considerable resource requirements for PIQ data collection and trace investigation, only high and moderate-risk movements were prioritized for detailed investigation in real-time during the outbreak period; however, a minimum dataset was collected for all movement regardless of risk classification. The planned approach is to conduct further investigations into nonhigh-risk indirect movement events for those IP identified as genetically connected, once the results of the GE are available, to optimize the use of available resources.

### 2.2. GE Methods and Tools

Individual viral segments (PB2, PB1, PA, HA, NP, NA, M, and NS) for the 305 viral genomes sequenced in this study were trimmed of regions flanking the open reading frames and concatenated. Concatenated whole-genome sequences were aligned using MAFFT v7.49 (totaling 13,112 nucleotides in length), and pairwise genetic distances were calculated with the R package APE: analyses of phylogenetics and evolution in R (v 5.7-1). The aligned sequences were used to construct a haplotype network using the median-joining method [20] implemented in the program PopART [21].

A maximum likelihood phylogenetic tree was estimated with the aligned A (H5N1) genomes using IQ-TREE v2.3.3 [22]. A separate partition was designated for each viral segment, allowing each to have its own model of nucleotide substitution, as determined by ModelFinder [23]. Node support for the resulting tree was assessed by 5000 ultrafast bootstrap replicates [24]. The bootstrap consensus tree was rooted and time-scaled using Augur-Refine from the Augur bioinformatics toolkit [25] with the options “--root best”, “--timetree”, “--no-covariance”, and “--stochastic-resolve”. Ancestral viral host and location estimates were reconstructed as discrete characters across all nodes of the time-scaled tree using Augur-traits from the Augur bioinformatics toolkit.

### 2.3. Transmission Network Analysis (TNA)

Retrospectively, a TNA was undertaken to better understand how A (H5N1) spread between IP. The objectives were (1) to identify the likely source (i.e., wild bird or contaminated environment versus premises-to-premises spread), and (2) to identify opportunities for lateral and local spread and possible chains of transmission. This was completed by analyzing the spatiotemporal data and hypotheses on transmission identified during the FE investigation, along with the taxonomic and phylogenetic information generated from the genomic analysis.

Epidemiological linkages were identified by reviewing each IP's known movement and contact events during the tracing period. Particular attention was given to movements that occurred in the 14 days before clinical signs for chickens and ducks, and 10 days for turkeys. Fourteen days correspond to the incubation period at the flock level according to WOAH [16]. Ten days was defined as the maximal incubation period for turkeys based on a review of mortality records on infected turkey operations. The time of introduction for turkey premises was less than 4 days, except for one flock at 9 days (CFIA, unpublished data, 2022). All movement events during the trace period, regardless of risk classification (i.e., high, moderate, low, and negligible) were retrospectively assessed (re-assessed for high and moderate risk events) for epidemiological linkages as a possible source of virus introduction to the IP or means of spread to another premises. This included all owner, workers, and service provider movements for persons and conveyances regardless of direct or indirect bird contact (e.g., feed deliveries, egg-pick up, catching crews, vaccination crews, etc.).

**Figure 1:**
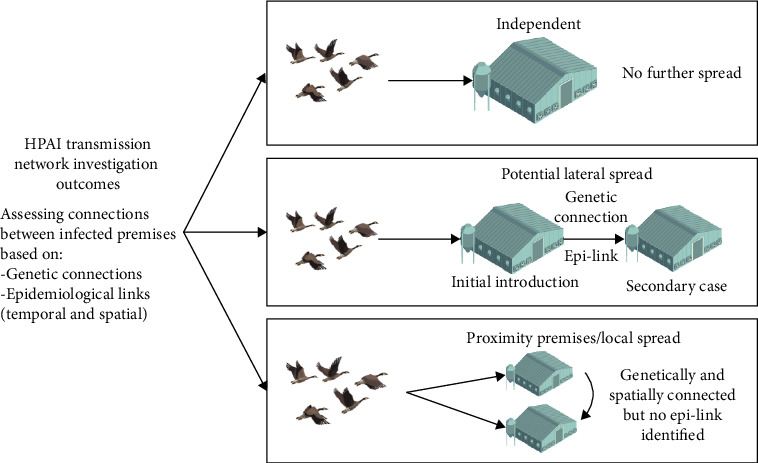
Transmission network analysis outcome interpretation.

The number of SNPs between the virus isolated from each premises was reviewed with consideration for the phylogenetic trees and median-joining networks to identify genetically connected premises for further investigation as either a source of A (H5N1) to another premises or a possible secondary case resulting from a between premises transmission events. Premises classified as genetically connected for retrospective tracing investigations due to the potential for lateral spread events had less than or equal to 15 SNP differences. This is consistent with findings from repeat sampling conducted on the same IP where SNP differences between viruses were between 0 and 15, with those at the higher end being farms with multiple bird species. In addition, geographic relationships were assessed by recording the shortest distance between affected barns on each IP and categorized into <1, 1–10, or >10 km. The TNA outcomes were categorized as an independent introduction, a possible lateral spread event, or a possible local spread event. For possible lateral and local spread events, the initial IP and secondary IP were established based on a temporal review of key dates, notably the start date of clinical signs, the date of movement restrictions, and the date when depopulation of all susceptible birds was complete.  Figure 1 illustrates the outcomes of the TNA.

For premises classified as independent introductions, premises were most likely infected through contact with, or environmental contamination from, wild birds; based on epidemiological and genetic information, these premises did not spread the virus to any other premises. For cases of lateral spread, an initial introduction on a premises may have led to one or more secondary cases due to epidemiological connections (such as movement of live animals or shared employes), which were temporally relevant. Premises were classified as a result of local spread (“proximity premises”) when the virus on two premises was genetically similar, and cases were linked geographically (within 10 km) and temporally (e.g., overlapping trace periods) but had no epidemiological linkages identified.

Outbreak data, including demographic data, event dates, and movement tracing investigation data, were stored in a Microsoft Access Database (Avian Influenza Operations Database version: AI 2022_National_Ops_v12.1 (2024-04-09). Data from waves 1–4 was manually extracted from the PIQ to generate hypotheses on risk or protective factors, saved in Microsoft Excel for Microsoft 365 MSO (Version 2402 Build 16.0.17328.20648) 64-bit, and visualized in PowerBI v2.122.746.0. Visualization and analysis of the descriptive epidemiology and TNA results were performed in Tableau (2024.2), and ArcGIS Pro v3.0.0 was used for mapping.

## 3. Results

The CFIA was notified on April 12, 2022, of sudden mortalities in a commercial broiler chicken premises located in the Regional District of North Okanagan (BC-IP01), and movement controls were placed. Approximately 53,000 30-day-old broilers were located in two barns on this commercial poultry premises. An increase in mortality was first observed on April 7, 2022, in the front quarter of 1 barn, with 166 dead birds observed by the producer. The birds in the second barn were reported as healthy. By April 12^th^, the mortality had increased to 4.0% of the total susceptible birds, and sampling was completed. Seven birds were sent for postmortem examination and showed severe congestion of the conjunctiva, head, skeletal muscles, trachea, lungs, and kidneys. Oropharyngeal swabs were collected and submitted to the BC CAHSN laboratory and the NCFAD on the same day. Samples were confirmed to be positive for the H5 subtype at NCFAD on April 13th. Depopulation was completed on April 15^th^, and a 10 km PCZ was established on April 16th. Virus isolation determined the sample to be A (H5N1) Subtype Gs/Gd with gene segments PB2 and NP belonging to the NA lineage and gene segments PB1, PA, HA, NA, M, and NS belonging to the Eurasian lineage. This virus was closely related to the original A (H5N1) virus identified in NL in December 2021, suggesting common ancestry with the Atlantic flyway A (H5N1) incursion rather than the Pacific flyway incursion.

Between April 2022 and December 2023, 158 domestic bird premises were infected with A (H5N1) in BC. The most affected sites were commercial poultry premises, accounting for 127 cases (80.4%), while 30 premises (19.0%) were noncommercial. One premise (0.6%) was a captive wild nonpoultry facility (considered noncommercial). Of the 30 noncommercial premises, 19 (63.3%) were classified as poultry premises, and 11(36.7%) were classified as nonpoultry premises. The 31 noncommercial premises infected with A (H5N1) housed between 1 and 260 birds. The 127 infected commercial poultry premises housed between 2127 and 317,238 birds. Of these, 14 premises contained multiple species, including either chickens and turkeys (7.1%) or chickens and ducks (3.9%). The remaining 113 premises included only a single species: 78 with chickens (61.4%), 13 with ducks (10.2%), 21 with turkeys (16.5%), and 1 with pigeons (0.8%).

**Figure 2:**
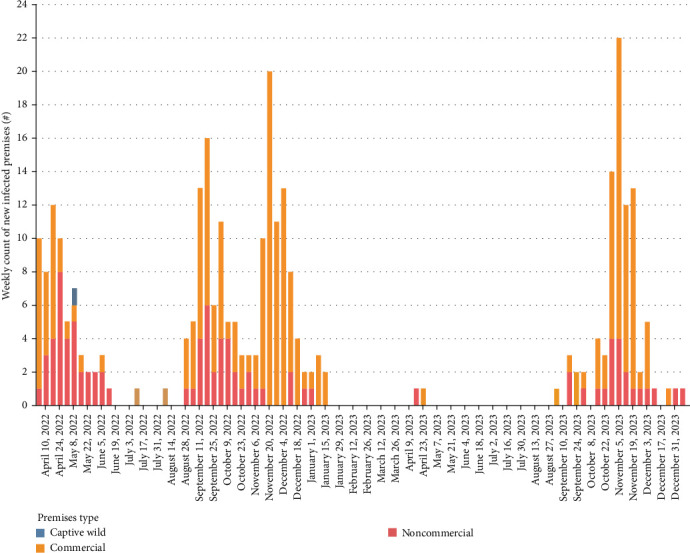
Epidemic curve of A(H5N1) cases by premises type, by week of first clinical signs (*n* = 158).

**Figure 3:**
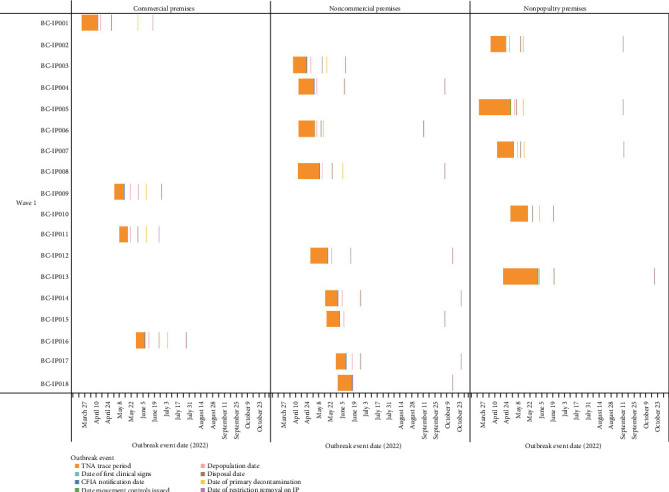
Timeline of trace period and outbreak events by premises type (Wave 1). The single captive wild bird infected premises (BC-IP010) detected in wave 1 is grouped with non-poultry.

**Figure 4:**
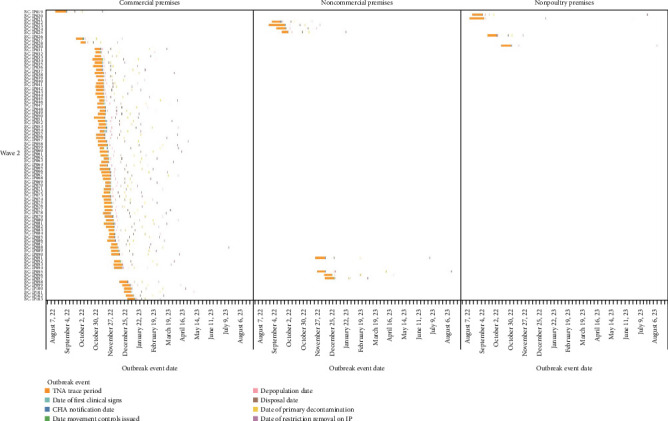
Timeline of trace period and outbreak events by premises type (Wave 2).

**Figure 5:**
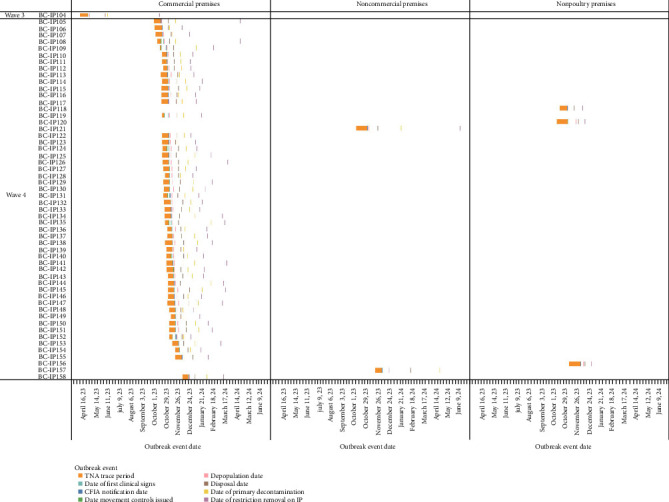
Timeline of trace period and outbreak events by premises type (Wave 3 and 4).

Of the 158 domestic poultry premises infected with A (H5N1) during this event, 127 (80.4%) premises were geographically located in the FV. The FV refers to the intensively farmed region of southwestern BC, primarily encompassing the FV regional district, where poultry and other agricultural operations are densely concentrated [26]. This included 125 of the commercial IP (98.4%) and two of the 30 noncommercial IP (6.7%). Of the 125 commercial IP in the FV, 121 (96.8%) were in one of three distinct geographical areas in the FV. This included 54 IP in the city of Abbotsford (44.6%), 52 IP in the city of Chilliwack (43.0%), and 15 IP in Langley Township (12.4%). The density of commercial poultry farms per developed square kilometer in these areas is estimated to be 1.00 in Abbotsford, 0.99 in Chilliwack, and 0.26 in Langley. During this HPAI event period, 17.2% of the commercial farms in Abbotsford, 24.6% in Chilliwack, and 15.8% were infected at least once with A (H5N1). Differences were noted between waves, with 54.8% of total commercial IP being in Abbotsford in wave 2 (fall of 2022) versus 55.1% being located in Chilliwack in wave 4 (fall of 2024).  Table 1 summarizes the number of IP by wave and premises type, and Figure 2 displays the temporal distribution of IP by premises type. Figures 3–5 visualize the timeline of outbreak events and trace periods for commercial, noncommercial and nonpoultry premises during each wave.

**Figure 6:**
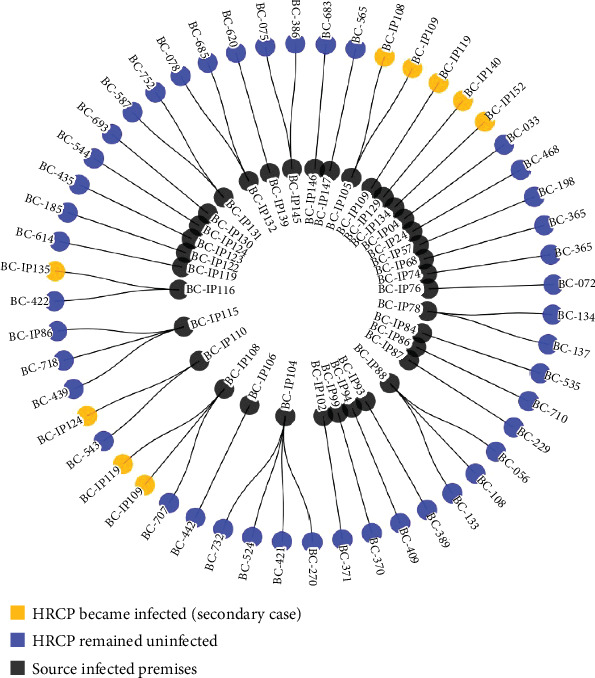
Outcomes of real-time investigation of high-risk contact premises (*n* = 50). Seven secondary cases were identified. BC-IP109 and BC-IP119 were identified as HRCP from more than one IP. BC-IP109 was also identified as a potential source of infection for BC-IP119.

During the event, 36 HRCP due to epidemiological linkages were identified. Each premises was issued a Declaration of Infected Place and a Requirement to Quarantine. Surveillance activities remained on these premises until 14 days after the last date of contact with the IP. Of these 36 HRCP, only one (2.8%) chicken layer premises became infected during the at-risk period. In wave 4 (fall 2023), the scope of the HRCP definition evolved to include premises located within 200 m of an IP. Fourteen (14) premises were identified as HRCP due to their spatial relationship with an IP; six (42.9%) of the 14 proximity premises became infected. This included three chicken layer premises, two chicken broiler premises, and one chicken broiler breeder.  Figure 6 shows the infection outcomes for the HRCP.

After each wave, phylogenetic trees, median-joining networks, and SNP differences were reviewed. Results supported the findings from the FE investigation. For the single hypothesized lateral transmission event and the six hypothesized local spread events due to proximity, the same virus genotype was identified on the source premises and associated HRCP.

Viruses from five (5) antigenically distinct clusters and 10 distinct genotypes were identified on the 158 IP during this period. For one premise, sequencing was incomplete or missing. This included seven premises (4.4%) with all gene segments belonging to Eurasian origin, nine premises (5.7%) with gene segments PB1 and PA belonging to NA lineage, 67 premises (42.4%) with gene segments PB2 and NP belonging to NA lineage, 71 premises (44.9%) with gene segments PB2, PB1, NP and NS belonging to NA lineage, and two premises (1.3%) with gene segments PB2, PB1, PA, NP and NS belonging to NA lineage. On one premise, two viruses were identified, both from distinct clusters.

**Figure 7:**
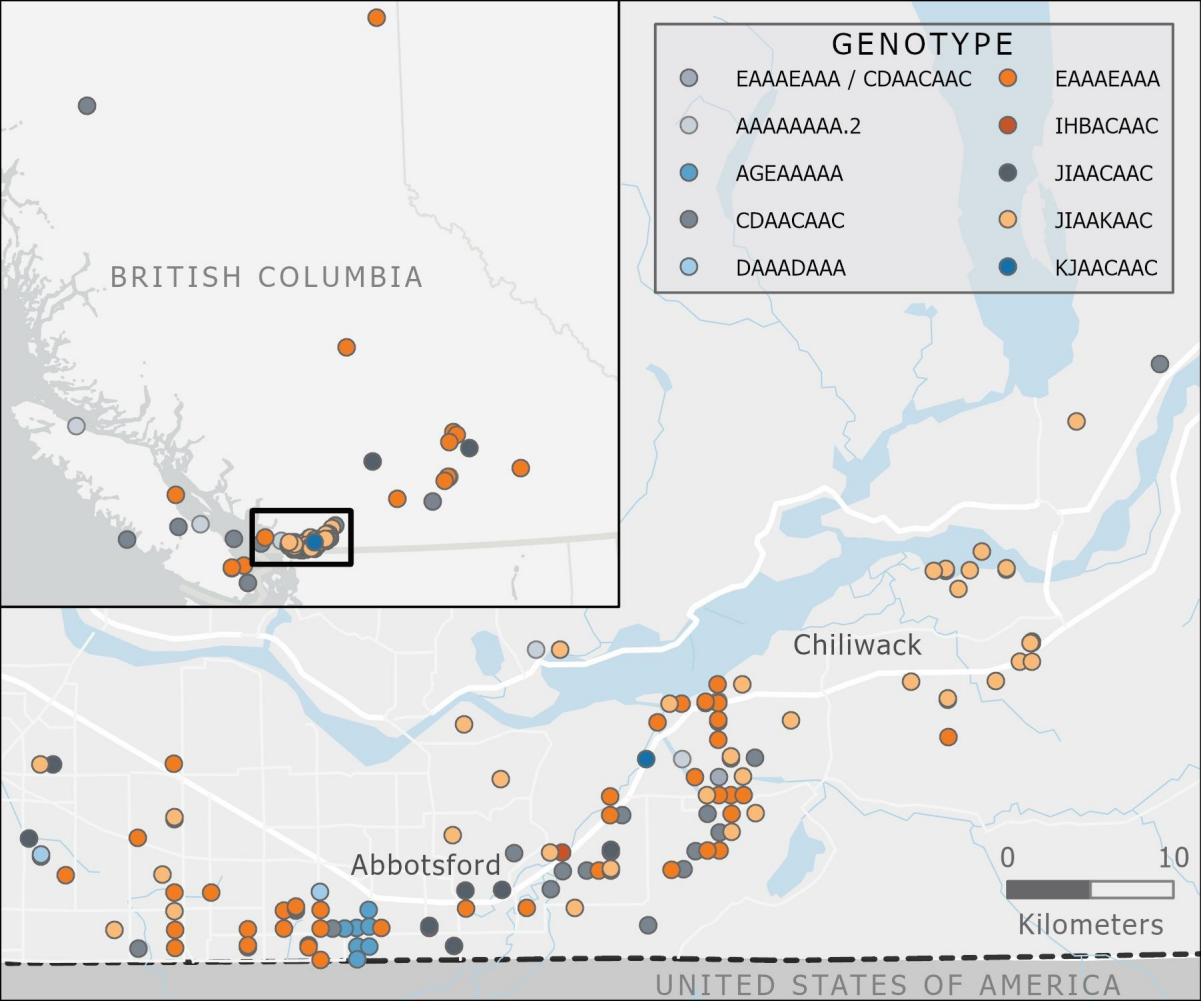
Location of A(H5N1) infected premises in British Columbia by genotype (*n* = 157).

**Figure 8:**
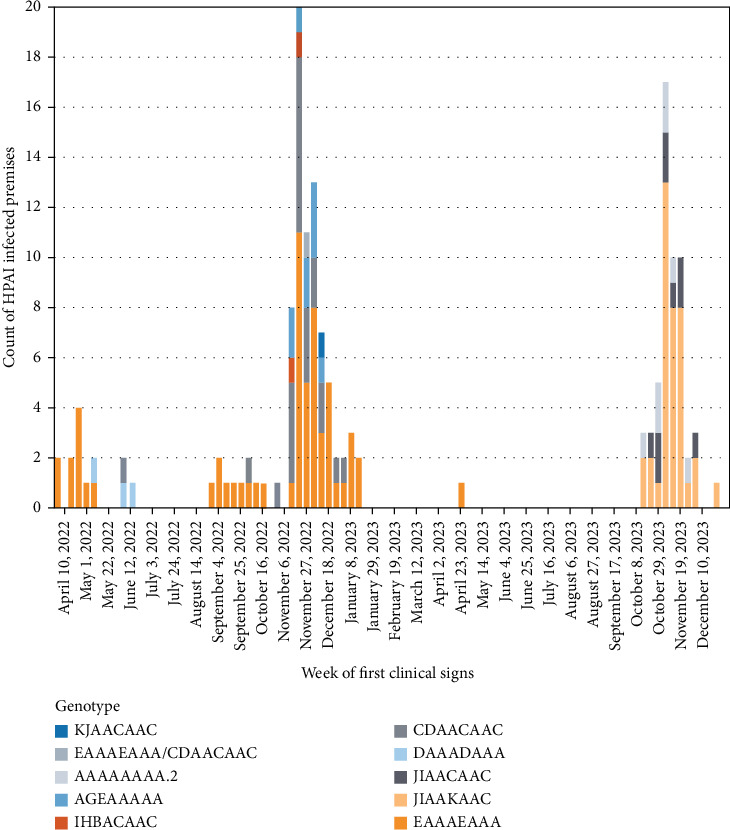
Epidemic curve of A (H5N1) cases by genotype, by week of first clinical signs (*n* = 157).

Virus isolated from most premises (89.9%) had a genetic origin from the Atlantic flyway introduction of A (H5N1); however, virus from nine premises (5.7%) had a genetic origin from the first Pacific flyway incursion, and virus from seven premises (4.4%) originated from the third Pacific flyway incursion.  Table 2 provides an overview of the virus characteristics, including origin, cluster, genotype, corresponding USDA genotype, and the count and proportion of IP.  Figure 7 maps the spatial distribution of IP by genotype, and Figure 8 presents the epi curve of IP by genotype, based on the start date of clinical signs.

In addition to the seven infected HRCP identified from the FE tracing and proximity premise investigations, multiple other genetically connected premises were identified. For these premises, all movement events during the trace period, regardless of the initial risk classification (i.e., high, moderate, low, and negligible), were retrospectively assessed for epidemiological linkages as a possible source of virus introduction to the IP or means of spread to other premises. The minimum spatial distance between the infected barns on each genetically connected IP was also determined.

During the real-time FE tracing of noncommercial premises, no HRCPs were identified. Virus from three of the five identified genetic clusters was detected on these premises. Of the 31 noncommercial premises, 27 (87.1%) were classified as independent introductions, while four premises (12.9%) were within 10 km of a genetically connected IP and classified as local spread. All infected noncommercial premises had birds with outdoor access.

Of the 127 infected commercial poultry premises, 21 premises (16.5%) were classified as independent introductions. One lateral transmission event and six local transmission events were hypothesized during commercial premises' real-time FE tracing. The lateral transmission event was associated with shared employes with direct bird contact on each premises. Results from the GE identified multiple other genetically connected premises. Each was further investigated to determine the potential for local or lateral spread.

**Figure 9:**
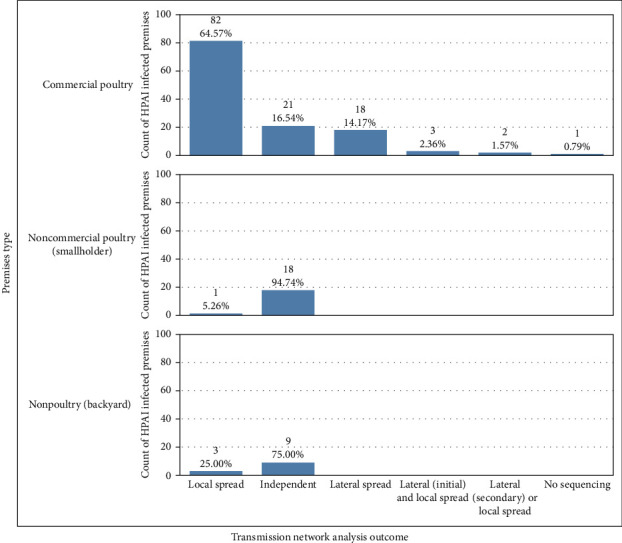
Distribution of infected premises by transmission network anlaysis outcome by premises type.

**Figure 10:**
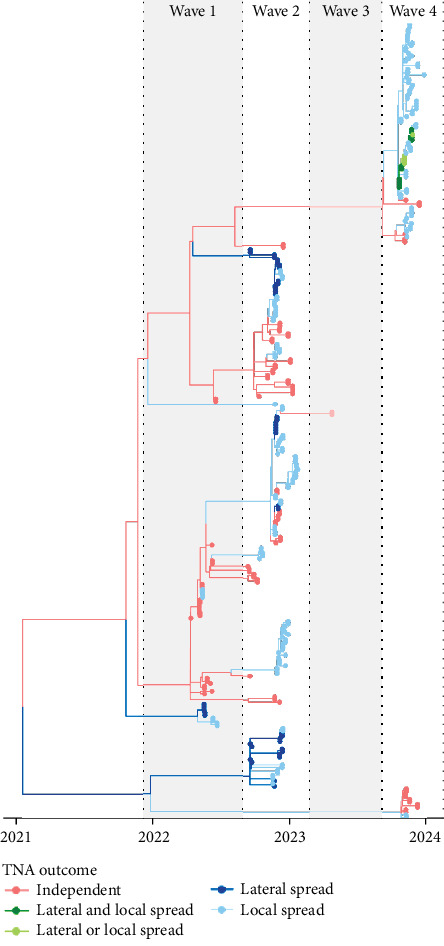
Time-scaled maximum-likelihood phylogenetic tree derived from 305 viral genomes collected from samples in British Columbia. Tree is colored by transmission network analysis outcome. Each point represents a virus isolate. Multiple samples may have been taken from an IP and because of this, each IP may be represented more than once.

In addition to the single lateral spread event/secondary case identified during the FE investigation, an additional 11 new potential lateral spread events were identified during the enhanced retrospective review of all movement events. One of these was identified as a proximity premises during the FE investigation, but the opportunity for lateral transmission had not been identified. This resulted in 11 additional potential lateral spread events (secondary cases) from 11 source premises, which had not been identified by the initial FE investigation. The hypothesized routes of lateral transmission included movement of live birds between IP (1), shared employes or equipment (3), shared service providers with direct bird contact (1), and shared service providers with indirect bird contact (6). Refer to Figure 9 for TNA outcomes stratified by premises type.  Figure 10 presents the phylogenetic tree highlighting genetic clustering and the TNA outcomes for each IP.

It should be noted that of the 12 premises with the potential to have resulted from lateral spread, all were located within 10 km of at least one genetically connected IP and of these, eight were located within 1 km, so the potential for local spread via a commonly contaminated environment cannot be excluded. No epidemiological connections to another IP were identified for the remaining 76 additional genetically connected IP identified through GE. However, local spread is suspected as all premises (100%) were located within 10 km of at least one genetically connected IP, and 35 (46.1%) were located within 1 km of at least one genetically connected IP.

There is also geographic overlap between poultry and dairy farms in BC, particularly in the FV. BC has 12% of commercial poultry farms in Canada [6], while dairy farms in BC account for less than 5% of all dairy farms [27]. HPAI is a reportable disease for all animal species. None of the sick cattle calls have detected A (H5N1). In addition, genotype B3.13 has never been detected in Canada. Genotype D1.1 was only detected in domestic birds in October 2024 (Wave 6). It is difficult to explain why spillover events to dairy cattle have not occurred in Canada, as these events are still poorly understood in the United States. However, it is important to note that both passive and active surveillance for HPAI in dairy cattle are in place in Canada [28].

During the event period (wave one to four), 19 commercial premises were infected twice, and one premise was infected three times. This included four premises infected for the second time during the fall of 2022 (wave 2), 15 for the second time, and one for the third time in the fall of 2023 (wave 4). The secondary and tertiary infections of these 19 premises were largely caused by the same viral genotype as the original infection, but were genetically distinct enough to support an independent viral introduction rather than reinfection from virus remaining on the farm. Species on premises with multiple introductions of A (H5N1) included seven with turkeys, seven with chickens, four with ducks, and one with both chickens and turkeys. In addition, two distinct viruses were isolated on two premises infected during wave two, indicating multiple introduction events.

For the route of introduction on commercial premises where lateral transmission was not identified, multiple potential risk factors were identified: known indirect contact with wild birds, proximity to wetland areas, barn structure/design (open or curtain-sided barns with lack of barn integrity), mortality disposed in areas with wildlife access, barns adjacent to agricultural fields, contact with untreated water, movement of birds within the premises during the critical period, lack of outerwear/clothing change when entering barns, and new bedding added to infected barn during the critical period.

## 4. Discussion

Distinguishing between local and lateral spread in genetically connected infected poultry premises is critical for understanding outbreak dynamics and implementing effective control measures, especially in regions like the FV of BC, where the high density of poultry farms presents unique challenges. Local spread associated with environmental contamination requires targeted containment strategies within affected zones, such as disinfection and access control. In contrast, lateral spread, typically resulting from biosecurity breaches, necessitates broader interventions like enhanced biosecurity protocols, traceability of shared resources, and stricter movement regulations. In high-density areas like the FV, the proximity of farms amplifies the risk of rapid local transmission and complicates efforts to distinguish between local and lateral pathways. This distinction is essential for prioritizing resources, as local spread demands containment within zones, while lateral spread requires extensive tracing and movement control. Furthermore, identifying lateral spread highlights systemic vulnerabilities in biosecurity, which can influence policy decisions and trade confidence. Understanding these spread mechanisms aids in defining disease-free zones, supporting zoning strategies, and modeling outbreak dynamics to predict and control future spread patterns.

This study was conducted in the FV, a region characterized by temperate climate, high farm density, and year-round wild bird presence due to its location along the Pacific flyway. These factors likely contributed to the region's disproportionate burden during Canada's largest recorded HPAI event. Although this context allowed for a detailed investigation of local spread dynamics, the findings may not be directly generalizable to other poultry production areas with differing ecological, environmental, or agricultural conditions. Future studies in varied regions are needed to determine whether similar transmission patterns and risks occur outside the FV.

Combining field and GE enabled a more comprehensive understanding of transmission networks. Field data on animal movements, premises management and biosecurity practices allowed for real-time tracing and risk categorization. However, FE relies on producer-reported information, which can be incomplete or biased due to recall error or reluctance to disclose noncompliance. These reporting gaps can lead to incomplete epidemiological linkages and may obscure the identification of true transmission pathways. In this study, the absence of reported contact or movement does not definitively rule out the potential for lateral transmission, particularly in densely populated regions where undocumented indirect exposures may occur. In contrast, GE provided high-resolution insights into viral evolution, confirmed or refuted suspected linkages, and identified reassortment events. The integration of both approaches allowed for developing detailed transmission maps and identifying genetic clusters consistent with observed outbreak patterns. Future outbreak response effects would benefit from embedding real-time genomic analysis into field investigations to optimize resource allocation and improve decision-making.

A key finding from the genomic analysis was the identification of mixed-origin viruses, with gene segments from both Eurasian and NA lineages. These resulted from reassortment events in wild bird populations, creating novel virus strains that subsequently entered domestic flocks. Although wild birds are recognized as a continuous source of virus introduction, the detection of genetically similar viruses within clusters of domestic premises, particularly within 10 km, underscores the importance of understanding within-region transmission. Eighty-three percent of commercial premises were located within an active PCZ at the onset of clinical signs, and proximity (≤200 m) to an IP was a stronger predictor of infection than direct epidemiological contact, suggesting that local environmental contamination played a substantial role in spread. Preliminary results from a spatial transmission kernel analysis of Wave 2 data suggest that transmission dynamics were highly localized and that environmental factors may play a role in disease spread. Further details will be available in a forthcoming study [29]. These results align with studies in North America, Europe, and Asia, which have shown that premises within a 10 km control zone had 3–32 times higher odds of infection [30–35].

Genomic data can confirm or refute hypotheses generated from field investigations. If FE suggests a link between premises, genomic data can provide genetic evidence to support or contradict this link, increasing the accuracy of outbreak investigations. The phylogenetic clustering of concatenated viral genomes and the median-joining phylogenetic network of the viruses supported, in all instances, the transmission network inferred by the FE HRCP investigations. However, there were multiple instances of common ownership, common management, or co-location of IP, which were not classified as an HRCP due to a lack of confirmed direct or indirect contact between the premises. There was often a lack of consensus on the risk posed by these premises; however, in many instances, the GE results identified distinct viruses, refuting the hypothesis of undisclosed contact events resulting in lateral spread.

GE can also identify genetic relationships between IP that were not identified during a field investigation as being connected, either spatially or as a result of epidemiological linkages. These premises can then be further investigated through more comprehensive retrospective tracing investigations of common service provider movements or other events not prioritized for tracing during the outbreak period. Multiple situations were observed where viruses from different IP had little or no genetic differences. Most of these premises were spatially related; however, a small number were located over 100 km apart. Despite a thorough investigation of all reported movement events and service provider logs, no epidemiological connections could be established. These situations suggest possible unreported direct or indirect epi-linkages or, for those premises in proximity, local spread through undocumented means, such as social networks, vectors, or windborne spread. For those premises which were very closely genetically connected (i.e., <5 SNP differences) but located very far apart a common wild bird source is most probable.

Most IP in this outbreak were commercial poultry operations, with a smaller number of noncommercial premises affected. This imbalance reflects the actual distribution of reported cases during the outbreak; however, it introduces important considerations regarding data interpretation. HPAI is a federally reportable disease, and all suspected or confirmed cases must be reported to the CFIA. To increase awareness and encourage reporting in noncommercial flocks, BC offers a Small Flock Health Program with free diagnostic services. Birds can be submitted via the flock veterinarians or directly by producers. Despite this requirement, underreporting may still occur [36]. Commercial producers have strong incentives to report as the compensation framework helps mitigate the economic burden of reporting for commercial operations and promotes greater compliance. If commercial cases are more likely to be detected and reported than noncommercial cases, this could potentially lead to an underestimation of the role of small flocks in HPAI transmission dynamics. Although noncommercial premises were included in this study, their small number and the potential for underreporting limit the ability to draw definitive conclusions regarding differences in infection risk and transmission patterns. Nevertheless, it is important to note that no epidemiological linkages between commercial and noncommercial IP were identified and no lateral spread events between noncommercial farms were identified, suggesting that these populations played an insignificant role in disease transmission.

While airborne spread was not directly assessed due to limitations in available weather data and microclimates in the FV, its potential role has been reported elsewhere [37–39]. Modeling studies suggest that windborne transmission over long distances is limited, but short-range airborne spread may be possible under specific conditions. For example, James et al. [40] detected viable virus only within 10 m of infected barns, Torremorell et al. [41] identified virus at 70 m, and Scoizec et al. [42] at up to 110 m. Considering the windborne risk, poultry farms within 200 m of an IP were quarantined and remained so until 14 days after the completion of carcasses and infected material disposal on the IP (capping of compost piles for farms doing composting). High-risk proximity farms were also tested for HPAI. This approach accounts for the potential risk of dispersing the virus after depopulating birds with carbon dioxide. Once birds are destroyed, barns must be ventilated to evacuate the gas, which could disperse the virus in the farm environment and increase the risk for surrounding farms. There are likely a combination of factors which could lead to infection of surrounding farms, including risky farm practices (e.g. movements of birds within the premises or split shipments to the slaughter plant) or biosecurity breaches during the exposure time window and the presence of infected higher risk species on the IP (e.g. ducks amplify the virus compared to other species). Given that many farms in the FV are located within several 100 m of one another, and that many of these proximity premises did not become infected with HPAI, further research is warranted to investigate the contribution of airborne transmission to local spread and identify management and biosecurity practices associated with spread of A (H5N1) between premises in close proximity.

The prominence of proximity-based spread also raises concerns about the effectiveness of on-farm biosecurity practices. A specific practice of interest is footwear disinfection. While 65% of commercial premises reported disinfecting boots, the methods varied widely, from powdered disinfectants to boot dips and sprays, and were not consistently applied. Footbaths are only effective if boots are visibly clean before immersion and sufficient contact time is maintained. In practice, these conditions are rarely met, and using footbaths without prior cleaning may increase contamination risk [43]. More effective alternatives include removing external footwear and donning dedicated clean boots after crossing a physical barrier, such as a bench [8, 44]. The type of hygiene barrier will have an impact on biosecurity compliance. Compliance is more likely when barn entry protocols are simple, visual, and enforceable [45]. The findings reinforce that biosecurity practices must not only be defined but also be feasible and adhered to under real-world conditions. However, the absence of comparable data from non-IP precluded formal case-control or cohort analyses. Without a comparison group, it is not possible to determine whether characteristics, such as wild bird presence or biosecurity measures were unique to infected sites or common across the broader poultry population. Future studies should incorporate data from infected and unaffected premises to strengthen the identification of statistically significant risk and protective factors.

Further research is needed to clarify the factors contributing to local spread, including environmental conditions, wind, wildlife activity, and farm-level biosecurity implementation. Investigating barn-level variables, such as structural integrity, access points, ventilation, and hygiene infrastructure will support more effective and enforceable disease prevention strategies. Integrating these insights into preparedness and response planning is essential to mitigate the economic, animal health, and public confidence impacts of HPAI outbreaks in high-risk regions.

## Figures and Tables

**Table 1:** Distribution of A (H5N1) cases by outbreak wave and premises type.

Outbreak wave	Poultry premises type		Case count	Case proportion (%)
Wave 1	Commercial	Poultry	4	2.53
	Noncommercial	Poultry	9	5.70
	NA	Nonpoultry	5	3.16

Wave 2	Commercial	poultry	73	46.20
	Noncommercial	poultry	8	5.06
	NA	Nonpoultry	4	2.53

Wave 3	Commercial	Poultry	1	0.63

Wave 4	Commercial	Poultry	49	31.01
	Noncommercial	Poultry	2	1.27
	NA	Nonpoultry	3	1.90

Total			158	100.00

**Table 2:** Distribution of virus characteristics by origin, cluster, and genotype.

Virus origin	Virus cluster	Virus genotype	USDA genotype	IP count	Proportion of IP (%)
Atlantic flyway	Gene segments PB2 and NP belonging to North American lineage and gene segments PB1, PA, HA, NA, M, and NS to Eurasian lineage	Not available	Null	4	2.53
		DAAADAAA	B2.1	3	1.90
		EAAAEAAA	B4.1	60	37.97
	Gene segments PB2, PB1, NP, and NS belonging to North American lineage and gene segments PA, HA, NA, and M to Eurasian lineage	CDAACAAC	B3.2	23	14.56
		JIAACAAC	B3.6	9	5.70
		JIAAKAAC	B3.10	38	24.05
		KJAACAAC	Not assigned	1	0.63
	Gene segments PB2, PB1, PA,NP, and NS belonging to North American lineage and gene segments HA, NA, and M to Eurasian lineage	IHBACAAC	Not assigned	2	1.27
	Incomplete or missing sequencing	Not available	Null	1	0.63
	Two viruses identified on the IP from different clusters	EAAAEAAA/CDAACAAC	B4.1/B3.2	1	0.63

Pacific flyway	All gene segments belonging to Eurasian lineage	AAAAAAAA.2	A3	7	4.43
	Gene segments PB1 and PA belonging to North American lineage and gene segments PB2, HA, NA, M, NS, and NP to Eurasian lineage	AGEAAAAA	Not assigned	9	5.70

			Total	158	100.00

## Data Availability

All genome sequences and associated metadata used in this study have been published in GISAID's EpiFlu database under the identifier EPI_SET_250206pw (DOI: https://doi.org/10.55876/gis8.250206pw). This dataset includes 305 individual A (H5N1) viral sequences collected in Canada between April 12, 2022, and December 25, 2023. To view the accession numbers, virus names, collection dates, originating and submitting laboratories, and contributing authors for each sequence, please visit the DOI link provided.
